# Protective role of exercise on breast cancer-related osteoporosis in women undergoing aromatase inhibitors: A narrative review

**DOI:** 10.1016/j.bonr.2024.101756

**Published:** 2024-03-25

**Authors:** Claudia Cerulli, Elisa Moretti, Elisa Grazioli, Gian Pietro Emerenziani, Arianna Murri, Eliana Tranchita, Carlo Minganti, Alessandra Di Cagno, Attilio Parisi

**Affiliations:** a Department of Movement, Human and Health Sciences, University of Rome Foro Italico, Piazza Lauro De Bosis, 15, 00135 Rome, Italy; b Department of Experimental and Clinical Medicine, “Magna Græcia” University, Viale Europa, 88100 Catanzaro, Italy

**Keywords:** Aromatase inhibitors, Breast cancer, Physical exercise, Osteoporosis prevention, Treatment

## Abstract

Hormone therapy following surgery reduces the risk of breast cancer (BC) recurrence and progression of hormone-sensitive BC, especially in postmenopausal women. Despite the antitumor efficacy of hormone therapy, particularly of aromatase inhibitors, they cause long-term side effects, mainly bone density reduction. Exercise can slow the rate of bone loss, which reduces the risk of fractures from osteoporosis, and could be an integrative treatment able to mitigate the BC treatment side effects positively impacting bone health. This narrative review aims to discuss studies on the effect of exercise on bone health in BC women undergoing aromatase inhibitors, highlighting the possible role of exercise as complementary to conventional therapies. Additionally, according to the literature revision, exercise practical applications to improve bone health in these patients are summarized.

## Highlights

• AIs increase risk of osteoporosis in Breast Cancer postmenopausal women.
• Exercise is an effective complementary therapy reducing risk related to AIs therapy.
• High-impact and multicomponent training are effective for osteogenic stimulation.
• Exercise can positively affect bone health in BC patients undergoing AIs therapy.

## Abbreviations

AIs: Aromatase Inhibitors
BC: Breast Cancer
BMD: Bone Mass Density
BPAQ: Baecke Physical Activity questionnaire
BPs: Biphosphonates
CON group: Control group
EX: Combined Training
FT: Flexibility Training
FU: Follow Up
HBEX: Home Based Exercise
HR: Heart Rate
INRAD: Italian National Regulatory Agency of Drugs
IU: International Unit
MVPA: Moderate-Vigorous Physical Activity
NTX: N-terminal propeptides of type I collagen
PA: Physical Activity
PEMFs: Pulsed Electromagnetic Field Stimulation
PW: Postmenopausal Women
PreMW: Premenopausal Women
QoL: Quality of Life
RCT: Randomized Controlled Trial
RM: Repetition Maximum
SERMs: Selective Extrogen Receptors Modulators
ST: Supplementation Therapy
SS: Supervised Exercise
TAM: Tamoxifene
WHO: Word Health Organizzation

## 1 Introduction

### 1.1 Breast cancer and osteoporosis

Breast cancer (BC) is the most common cancer among women worldwide. Five hundred and thirty-one thousand new cases of BC in Europe were recorded in 2020, and it is expected an increase up to 586.000 in 2040 (GCO, 2020). Worldwide, osteoporosis is estimated to affect 200 million women - approximately one-tenth of women aged 60, one-fifth of women aged 70, two-fifths of women aged 80 and two-thirds of women aged 90 (IOF). These two disorders are common in postmenopausal women, and they share a common underlying factor: estrogenic. Moreover, high incidence of osteoporosis has been reported in breast cancer patients due to early menopause triggered by adjuvant treatment and temporary ovarian function suppression (Go et al., 2020).

About 70–80 % of all breast tumors are considered Hormone Receptor-positive (HR+). Hormone Receptor-positive breast cancer (HR+ BC) may be positive for estrogen receptors (ER+), progesterone receptors (PR+), or both (ER+ and PR+) (Walsh et al., 2020). HR+ BC usually must be treated with Hormone Therapies. Currently, Aromatase Inhibitors (AIs) represent the standard HR+ BC care, mainly in Postmenopausal Women (PW) and in those at high risk of relapses (Nardin et al., 2020). Despite their antitumor efficacy, AIs are related to several long-term side effects like musculoskeletal disorders (i.e. arthralgia), secondary osteoporosis as a direct consequence of the hypo-estrogenic condition derived from the aromatase suppression (Tenti et al., 2020) and to anemia, which reduces muscle strength and induces a high inflammatory status (Natalucci et al., 2021). Focusing on the topic of this narrative review, it is necessary to explain why secondary osteoporosis occurs after AIs treatments leading to loss of Bone Mineral Density (BMD).

Secondary osteoporosis is described as low bone mass with bone micro architectural alterations, which could lead to fragility fractures in the presence of an existing disease or pharmacological therapy (Mirza and Canalis, 2015). Therefore, it is a consequence of pathological conditions such as cancer (i.e. breast and prostate), kidney failure, or it may result after prolonged use of pharmacological therapy, specifically, hormone therapies, Chemotherapies, and Radiotherapies increase the risk of secondary osteoporosis, particularly in women with HR+ BC (Nakamura, 2005; Watts et al., 2010; ESCEO, 2012).

The bone homeostasis develops on three levels: a ‘macro’ level regulating the bone balance through different systemic hormones such as estrogen, androgen, calcitonin and parathyroid hormone; a ‘*meso*’ level supported by the mechanical load of forces and the gravitational force acting on the bone stimulating osteogenesis; a ‘micro’ level resulting from balance of construction and reabsorption of bone micro-architecture performed by osteoclasts and osteoblasts activity respectively (Gennari et al., 2011). To preserve a healthy bone, it is necessary to act on these three levels. Evidence suggests that apply mechanical forces on bone, through proper exercise, could be a feasible approach to induce bone health in HR+ BC women (Hutzler and Sherrill, 2007; Foglietta et al., 2017). Not surprisingly, adapted exercise is increasingly being studied in both osteoporosis and oncological pathologies, and more specifically in BC as a complementary therapy. Adapted exercise is a well-structured exercise with fixed parameters like: frequency, intensity, time, and type (FITT parameters) (Hutzler and Sherrill, 2007).

### 1.2 Effect of aromatase inhibitor on bone health loss in HR + BC patients

AIs are mostly used in post-menopausal women with ER + BC, because during the Phase III of clinical trials they shown improved anticancer efficacy.

**Fig. 1:**
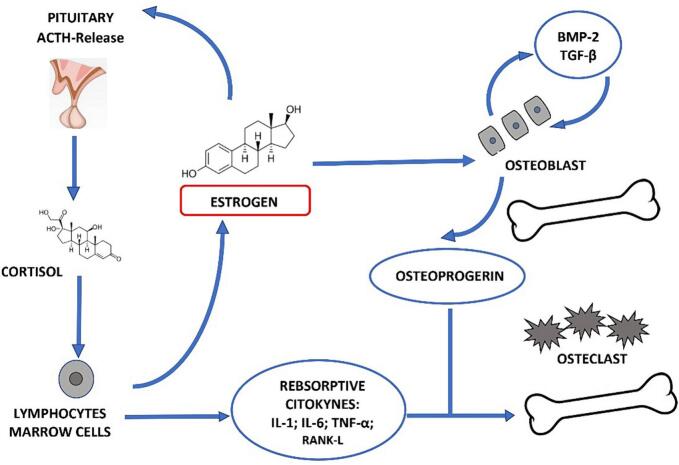
The estrogen role in bone metabolism. Abbreviation Il, interleukin; TNF-α, Tumor necrosis factor alpha. (COLOUR SHOULD BE USED FOR THIS FIGURE).

According to the American Society of Clinical Oncology clinical practice guidelines, AIs are recommended for at least five years after surgery or can be used as a sequential strategy after 2–3 years of Tamoxifen, (Burstein et al., 2010; Tenti et al., 2020). More in-depth, it is important to explain the link between AI's use and BMD loss. Firstly, the key role of estrogen in bone health must be pointed out: estrogen plays a central role in bone resorption being related to osteoblasts and osteoclasts activity. Osteoblast secretes both osteoprogerine (OPG) and the Receptor Activator of Nuclear Factor-KB Ligand (RANKL) which leads to cell differentiation into mature osteoclasts that stimulatates bone resorption. In addition, OPG inhibits osteoclastic. The sex hormone deficiency occurring during menopause, or worsen due to AIs use, stimulates T cells to secrete tumor necrosis factor alpha (TNF-α) and RANKL activating osteoclasts causing bone resorption (Andersson et al., 2005; Suskin and Shapiro, 2018) (Fig. 1).

Evidence highlights an increased prevalence of fractures in BC women or BC survivors, with or without metastases, in comparison with the healthy female population due to the prolonged use of hormone therapies particularly AIs (Kanis et al., 1999; Chen, 2005; Camacho et al., 2020). It is possible to suggest that AIs treatment leads to higher bone mass loss and risk of fractures, particularly of hip and lumbar spine in HR+ BC PW. To date, several randomized trial studies evaluated the effects of pharmacological treatment in HR+ BC patients undergoing hormone therapies to prevent bone loss. Currently approved treatments are bisphosphonates, Denosumab (anti-receptor activator of nuclear factor-kappaB ligand antibody) and Sclerostin Inhibitor. These treatments seem to be potentially preventive in secondary osteoporosis caused by hormone therapies, in particular AIs therapy (Folkestad et al., 2009; Camacho et al., 2020). Thus, starting pharmacological therapies with zoledronate, bisphosphonates or denosumab seems essential (Camacho et al., 2020) in BC patients. However, a discussion of bone modifying agents in the setting of early stage breast cancer treated with AI therapy is beyond the scope of this review, which aim to identify the role of exercise in the prevention and control of bone loss in patient affected by HR + BC.

## 2 Role of exercise in breast cancer and osteoporosis

Nowadays, scientific literature reports the benefit of exercise sustaining the hallmarks of health, preventing the onset of chronic degenerative diseases, conferring multiple health benefits and improved survival. This is due to the adaptation of multiple tissues (e.g. muscle and bone) and organs (e.g. heart, lungs). Exercise appears to be a valuable protective strategy to maintain health in response to stress, and is considered a non-pharmacological pill for patients with certain comorbidities and diseases (Qiu et al., 2023). In literature, it is now clear that physical exercise plays a fundamental role in the prevention and maintenance of bone health, and is increasingly recommended as a complementary therapy in osteoporosis. The types of activities suggested by the guidelines (Brooke-Wavell et al., 2022; Bae et al., 2023) are reported in Table 1 below.

**Table 1:** Physical activity guidelines for osteoporosis prevention (Brooke-Wavell et al., 2022; Bae et al., 2023).

Exercise type	Purpose	Frequency	Recommended activities
Impact training	Maximize bone strength	3/5 days/week	- Stamping - Jogging - Low-level jumping or hopping – Dancing – Brisk walking – Stair climbing
Resistance training	Maximize bone strength	2/3 days/week	- Upper body exercises using weight stimulating muscles which origin and insert to the spine. -Lower body exercises using weight stimulating muscles which origin and insert to the hip.
Body weight training	Maximize bone strength	2/3 days/week	- Upper body exercises using elastic band or the body weight stimulating muscles which origin and insert to the spine. -Lower body exercises using elastic band or the body weight stimulating muscles which origin and insert to the hip.
Balance training	Reduce falls	3/5 days/week	- Thai-Chi – pilates - yoga

Not surprising the World Health Organization, recommend to people who have had a breast cancer diagnosis to carry out the same amount of physical activity as the general population. Little is known about the specific type, duration and amount of exercise needed during and after breast cancer diagnosis, despite some guidelines have been drafted over time (Panel, 2010; Rock et al., 2012; Schmitz et al., 2019) (Table 2).

**Table 2:** Physical activity guidelines for breast cancer patients. Abbreviation: ACSM, American College of Sport Medicine; ACS, American Cancer Society; ACoR, American Congress of Rehabilitation Medicine; Min, Minutes; Reps, Repetitions.

Societies	Parameters	Aerobic	Resistance	Stretching
ACSM (Panel, 2010)	Frequency	3 day/week	2–3 day/week	3 day/week
	Intensity	Moderate	Moderate - Vigorous	slight discomfort no pain
	Time	30–60 min	8/15 reps for 2/4 sets for the major muscle's groups	20–40 min
	Duration	at least 8 weeks	at least 8 weeks	at least 8 weeks
ACS (Schmitz et al., 2019)	Frequency	3–7 day/week	2 day/week	2 day/week
	Intensity	1)Moderate 2) Vigorous	no specific indication	no specific indication
	Time	1)150–300 min/week 2)75–100 min/week	30–60 min	10–30 min
	Duration	no specific indication	no specific indication	no specific indication
ACoR (Rock et al., 2012)	Frequency	3 day/week	2 day/week	no specific indication
	Intensity	Moderate intensity	no specific indication	no specific indication
	Time	Up to 30–60 min/day	20–30 min	no specific indication
	Duration	no specific indication	no specific indication	no specific indication

However, in scientific literature, adapted exercise is currently suggested in all phases and types of BC (Dimauro et al., 2021; Tranchita et al., 2022; Grazioli et al., 2020). There are implications to more specific research on the best type of adapted exercise suggested for the different types of BC. To date, interventions to improve patients' health status in ER + BC women undergoing hormone therapies are important, since it is necessary to increase BC patients' quality of life and to neutralize therapies' side effects such as secondary osteoporosis (Cormie et al., 2018; Campbell et al., 2019). Therefore, despite is well known the preventive and protective role of adapted exercise in the osteoporosis process and management, it is still unclear what type and dose of exercise is most appropriate to prevent and limit secondary osteoporosis onset in HR+ BC. Thus, this narrative review aims firstly to analyze the effect of exercise on BMD and bone health in women with ER + BC undergoing AIs treatment; secondly, the review aims to outline preliminary practical applications on the most effective protocols in preventing BMD loss in these patients.

### 2.1 Complementary intervention for osteoporosis in BC patients

Complementary treatments and interventions for osteoporosis, like calcium and vitamin D supplementation and/or exercise may have a positive impact on bone health in post-menopausal woman with HR+ BC, despite the issue is still controversial and need attention (Polzonetti et al., 2020; Tański et al., 2021; Datta and Schwartz, 2013). In fact, calcium and vitamin D supplementations alone seem not enough to prevent and treat bone loss in BC patients, but there is a lack of evidence on this specific population (Datta and Schwartz, 2013). To date, supplementation of calcium and vitamin D plays a synergistic role in association with pharmacological antiresorptive drugs therapy by reducing the fracture risk and preventing hypocalcemia (Diana et al., 2021). Moreover, a review published in 2021 reported that exercise could induce an anabolic or homeostatic effect in the bone tissue through mechano-transduction in post-menopausal woman (Murri et al., 2021). Therefore, gravitational-type activities, such as jumping and running may cause bone stress leading to subsequent mechano-transduction and therefore increased BMD. Unfortunately, is still unclear the most effective type, frequency, and volume of exercise to improve bone health in BC women undergoing hormon therapies.

## 3 Materials and methods

**Fig. 2:**
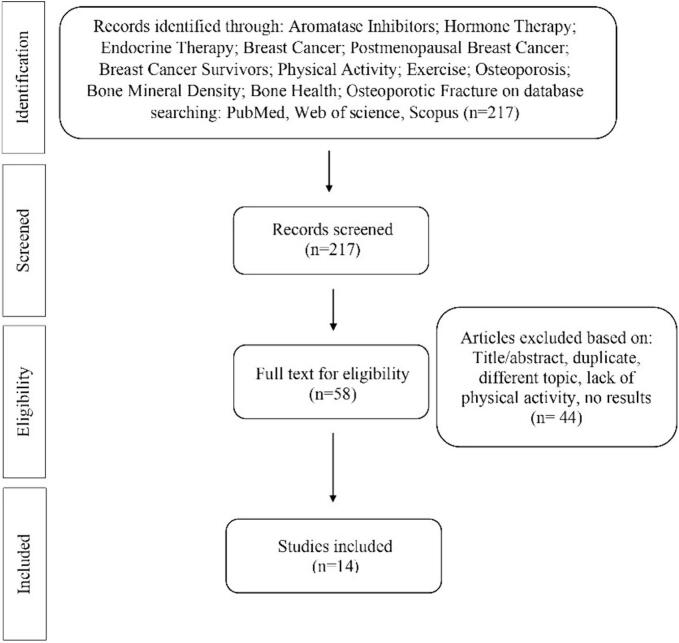
Research Strategy. (COLOUR SHOULD NOT BE USED FOR THIS FIGURE).

The purpose of this article is to narratively review studies highlighting the role of physical exercise on bone loss induced by cancer-related treatments, in particular AIs, in ER + BC patients. Three online databases, PubMed, Google Scholar, and MEDLINE were used by two authors, and the search was limited to peer-reviewed journals written in English. The narrative review includes studies published from 2003 to 2023. The research keywords were: Aromatase Inhibitors; Aromatase; Hormone Therapy; Endocrine Therapy; Breast Cancer; Breast Neoplasm; Postmenopausal Breast Cancer; Breast Cancer Survivors; Physical Activity; Exercise; Training; Osteoporosis; Bone; Bone Mineral density; Bone turnover; Bone loss; Osteoporotic fracture. A manual search of the reference lists in the studies found in the computerized search was also conducted. 217 studies were found. Once the duplicates were removed, 58 studies remained, and 44 studies did not match the above-mentioned criteria. At the end, 14 studies have been selected in the review. The research strategy is summarized in Fig. 2.

## 4 Results

As reported in Table 3 the studies were divided in 3 sections according to the main outcome analyzed:

• preventing fracture risk and loss of BMD
• modulating bone metabolism markers
• the effect of exercise combined with osteoporosis supplementation therapy of pharmacological antiresorptive therapy

**Table 3:** Effect of exercise on Bone Health in Breast Cancer patients undergoing AIs treatments. Abbreviation: PW, Postmenopausal Women; PreMW, Premenopausal Women; BC, Breast Cancer; RCT, Randomized Control Trial; BMD, Bone Mass Density, d, day; w, week; s, seconds; min, minutes; rep, repetitions; mo, months; ST, Supplementation Therapy; EX, Combined Training; SS, supervised; HBEX, Home Based Exercise; HR, Heart Rate; CON, Control; PEMFs, Pulsed Electromagnetic Field stimulation; PhT, Pharmacological Therapy; FU, Follow Up; FT, Flexibility Training; PA, Physical Activity; TAM, Tamoxifene; AIs, Aromatase Inibitors;

Author	Type	Sample	Treatments	Intervention	Results
The effect of exercise in preventing fracture risk and loss of BMD					
Irwin et al. (2009)	RCT	PW (n = 75) Age:47–66 SS-HBEX (n = 37) CON (n = 38)	AIs TAM Other Therapies	6-mo SS-HBEX: Aerobic Training, 3 d/w and 2 d/w, 150 min/w CON: No intervention	SS-HBEX ↔ BMD CON ↓ BMD
Saarto et al. (2012)	RCT	PreMW (n = 229) Age:35–58 SS-HBEX (n = 124) CON (n = 105) PW (n = 269) Age:46–68 SS-HBEX (n = 138) CON (n = 131)	AIs ≥ 4-mo TAM ≥ 4-mo Other Therapies	12-mo SS-HBEX SS: Step Aerobics Training and Impact Training, 1 d/w, 60 min at 118 bpm (14–16 RPE) HBEX: Endurance training and Impact training, 2–3 d/w CON: No intervention	PreMW SS-HBEX and CON ↓Lumbar spine BMD ↑Femoral Neck SS-HBEX vs CON ↔ Femoral neck BMD PW SS-HBEX and CON ↓Lumbar spine, Femoral neck BMD PreMP and PW SS-HBEX ↑Lean Mass
Nikander et al. (2012)	RCT	Pre and PW (n = 77) Age:35–68 SS-HBEX (n = 37) CON (n = 40)	AIs TAM Other Therapies	12-mo SS-HBEX: Unsupervised 1 d/w Impact Aerobics Training, 40 min at 80 % HRmax, 100–150 jumps, hops and leaps, 2–3 d/w unsupervised Impact Aerobics Training, 100 hops, leaps and jumps Endurance training, 150 min/w at 14–16 RPE CON: No intervention	PW SS-HBEX and CON Bone loss SS-HBEX vs CON ↑Bone structural strength SS-HBEX ↑Femoral Neck and Tibial Diaphysis BM Distribution PreMW SS-HBEX ↔BMD Femoral neck
Vehmanen et al. (2021)	RCT	PreMW and PW (n = 444) Age:35–68		FU-60-mo Saarto et al. (2012)	PreMW SS-HBEX ↔ Femoral neck, Total hip BMD for 12-mo 24-mo SS-HBEX and CON ↓ Femoral neck, Total hip, Lumbar spine BMD at 60-mo PW SS-HBEX and CON ↓Femoral neck, Total hip, Lumbar spine BMD
Kwan et al. (2023)	Prospective RCT	Pre and PW (n = 2152) Age (28–94)		6-mo Self-reported PA with Arizona Activity Frequency Questionnaire to evaluate PA levels 6-mo before and 6-mo after BC diagnosis	6-mo Before and After BC diagnosis of overall moderate-vigorous PA No association with Fracture Risk 6-mo Before BC diagnosis of none or infrequent overall moderate-vigorous PA ↑Osteoporosis risk 6-mo Before and After BC diagnosis of <150 min/w of Aerobic Training ↑Fracture risk

The effect of exercise in modulating bone metabolism markers					
Knobf et al. (2008)	Pilot Study	PeriMPW and PW (n = 26) Age: 45–58	AIs TAM Other Therapies	6-mo Walking Training with weight belt and backpack, 3 d/w, 45 min, at 65–75%HR, 5 lb	↔ Osteocalcin, NTxserum ↔ BMD
Winters-Stone et al. (2011)	Single-Blind RCT	PW (n = 106) Age:≥50 SS-HBEX (n = 52) FT (n = 54)	AIs or SERM Other Therapies Bisphosphonates	12-mo SS-HBEX SS-HBEX: Unsupervised Resistance Training and Impact Training, 2 d/w 45-60 min at 8–10 RM 1-2 × 8–12rep and 10 % of BW 6x10rep and supervised Resistance Training and Impact Training, 1 d/w FT: Stretching and Relaxation EX	SS-HBEX vs FT ↔Lumbar Spine BMD ↑Osteocalcin ↑Deoxypyrodinoline SS-HBEX vs FT under AIs ↑FFM ↓Fracture Risk
De Paulo et al. (2018)	Single-Blind RCT	PW (n = 36) Age:50–80 EX (n = 18) FT (n = 18)		9-mo EX: Combined Training, 3d/w, 40 min at 75 % 1-RM, 3 × 8-10rep and 30 min at 75–80%HRmax FT: 2d/w, 45 min, 10–15 s each position	EX vs FT ↑ Osteocalcin ↓FM ↔BMD
Saito et al. (2020)	Observational Study	PMW (n = 53) Age:59–76		Self-reported PA with BAECKE PA questionnaire to assess daily PA levels and Accelerometer parameters	Light PA ↑ Bone Formation Markers ↓ Bone Resorption Markers

The effect of exercise combined with osteoporosis supplementation therapy					
Waltman et al. (2010)	RCT	PW (n = 233) Age:35–75 EX-ST (n = 110) CON-ST (n = 113)	AIs TAM	24-mo ST: 1.200 mg Ca, 400 IU Vitamin D and 35 mg/w PhT: Biphosphonates EX-ST: 9-mo - Resistance Training at home, 2 d/w, 30-45 min 15-mo - Resistance Training, 2 × 8–12 rep + ST and PhT CON-ST+ PhT: ST + PhT only	EX-ST ↑ Total hip, Femoral Neck, Spine and Total Radius BMD ↓Alkphase B ↓ NTx serum CON-ST ↑ Total hip and Spine BMD and femoral neck ↓Alkphase B ↓ NTx serum ↓BMD Radius EX-ST vs CON-ST ↑ BMD Total hip, Femoral Neck, Spine and Total Radius BMD But not statistically significant
Knobf et al. (2016)	RCT	PeriMP and/or Early-PW (n = 150) Age:45–61 SS-HBEX-ST (n = 76) CON (n = 74)	AIs TAM Without AIs Without TAM Other Therapies	12-mo ST: 1200 mg Ca, 400 IU Vitamin D SS-HBEX-ST: Combined Training, 6-mo, 3 d/w, 30 min at 65–70%HRmax and 5EX at 70 %1-RM, 1 × 8 rep, Combined Training, from 7-mo to 12-mo, 3 d/w, 30 min at 65–70%HRmax and 5EX at 70 %1-RM, 1 × 8 rep+ ST CON-ST Recommended 30 min of moderate intensity activity most days of the week+ ST	SS-HBEX-ST under AIs ↓Lumbar spine and Greater Trochater BMD ↑ Osteocalcin CON under AIs ↓ Femoral neck BMD ↑Bone resorption SS-HBEX-ST under AIs vs CON under AIs ↑Bone resorption ↑Bone formation SS-HBEX-ST under TAM ↓ Femoral neck ↓ Bone resorption SS-HBEX-ST and CON under TAM and SS-HBEX-ST and CON Without AIs or Without TAM ↔Lumbar spine, Greater trochanter BMD - SS-HBEX-ST Without AIs or Without TAM vs CON Without AIs or Without TAM ↑Bone resorption
Kim et al. (2016)	RCT	PW Osteopenic (n = 43) Age:50–63 HBEX-ST (n = 23) CON-ST (n = 20)	AIs or SERM Other Therapies	6-mo ST: 500 mg Ca, 1000 IU Vitamin D HBEX-ST: 3 d/w unsupervised Walking Training, 150 min/w at 12–13 RPE, 2–3 d/w Resistance Training unsupervised 2 × 8-10rep with Progressive Resistance TheraBand+ ST CON-ST ST only	HBEX-ST and CON-ST ↑NTx and 25-hidroxyvitaminD CON-ST vs HBEX-ST ↓Lumbar spine, Total Hip But not statistically significance
Thomas et al. (2017)	RCT	PW (n = 121) Age: 55–69 SS-HBEX (n = 61) CON (n = 60) BHs-SS-HBEX (n = 11) BHs -CON (n = 9)	AIs ≥ 6-mo Other Therapies	12-mo PhT: Biphosphonates -FT-SS-HBEX: Resistance Training, 2d/w, 6 EX, 3 × 8–12rep Aerobic Training, 150 min/w at 60–80%HRmax PhT- CON: No intervention	PhT- SS-HBEX vs PhT-CON ↑FFM↓FM No difference in BMD PhT- SS-HBEX vs PhT-CON ↑ BMD
Ashem et al. (2019)	RCT	Osteoporotic (n = 45) Age (45–55) EX-ST (n = 15) PEMFs-ST (n = 15) CON-ST (n = 15)		3-mo ST: 11.2 mg Ca, 0.5 mg/day Vitamin D EX-ST EX: Treadmill, weight­bearing, 3d/w, 30 min at Moderate intensity PEMFs-ST PEMFs: Electromagnetic field, 3d/w, 30 min at 72 Hz + ST CON-ST ST only	PEMFs-ST > EX and CON ↑ Lumbar spine BMD EX > CON ↑ Lumbar spine BMD PEMFs-ST, EX and CON ↑ Lumbar spine BMD BMD Lumbar Spine CON < EX < PEMFs-ST

In the first section two studies described combined training protocols (Saarto et al., 2012; Vehmanen et al., 2021), one study an aerobic training (Irwin et al., 2009), one study a multicomponent exercise protocol (Nikander et al., 2012), and one manuscript evaluated the level of physical activity through a questionnaire (Kwan et al., 2023). In the second section, three studies reported combined training (Knobf et al., 2008; Knobf et al., 2016; De Paulo et al., 2018), one study a multicomponent exercise protocol (Winters-Stone et al., 2011), and one manuscript evaluated the level of physical activity through a questionnaire (Saito et al., 2020). In the third section, two studies evaluated combined training (Thomas et al., 2017; Knobf et al., 2016; Kim et al., 2016), one manuscript a resistance training (Waltman et al., 2010), and one manuscript a pulsed electromagnetic stimulus (Ashem et al., 2019). What figured out is that, there is still considerable research to do on the most appropriate exercise in the prevention of secondary osteoporosis in women undergoing AIs therapy (Waqas et al., 2021). To date, published studies are conflicting, while systematic review has evidenced that resistance training or impact exercises did not improve BMD at the lumbar spine, femoral neck, or total hip in women undergoing AIs therapy (Fornusek and Kilbreath, 2017). However, a Randomized Control trial (RCT) has shown an increase in BMD at the femoral neck but not at the lumbar spine after 12 weeks of supervised weight-bearing jumping exercises and circuit training in BC survivors after adjuvant therapy (Saarto et al., 2012).

## 5 Discussion

The narrative review, as reported in Table 3, highlights that physical exercise is effective in the prevention of secondary osteoporosis in BC patients by acting on three different levels: in preventing BMD loss, modulating biomarkers of bone metabolism, and in assisting the effects of pharmacological supplementation.

Indeed, studies that assessed physical activity levels in premenopausal and postmenopausal women undergoing treatment have shown that performing moderate to vigorous physical activity for 150 min per week has a protective effect on osteoporosis fracture, especially in older women (Irwin et al., 2009; Kwan et al., 2023). Interestingly, if exercise levels are below 50 min per week, there is no benefit in BMD loss and fracture prevention (Irwin et al., 2009); therefore it is crucial to prescribe a correct exercise dosage to benefits bone health. This should be stated in the guidelines, that so far reported mainly the days per week. Exercise protocols with high-impact or strength training have showed great efficacy in reducing BMD loss at the femur, lumbar spine and tibia, after 1 year of exercise intervention, in women treated with Ais (Saarto et al., 2012; Nikander et al., 2012; Vehmanen et al., 2021). In particular, it has been showed by Nikander et al., a higher bone structural strength maintenance and a better bone distribution at the femoral neck in women who practiced physical activity compared to those inactive (Nikander et al., 2012). Women enrolled in the control group, on the other hand, have showed a loss of BMD in all the sites analyzed after 1 year. It is important to underline that training may maintain the protective effects after 24 months, but they seem gradually reduced after 36 and 60 months after the end of the exercise protocol proposed. It evidences that the crucial point to benefit from physical exercise is the continuity, and that it can be a protective tool for bone loss if it is practiced regularly (Kim et al., 2016). Overall, exercise increases lean mass (Winters-Stone et al., 2011; Saarto et al., 2012; Thomas et al., 2017) a determinant in preventing the risk of falls, fractures and loss of BMD. Not only because a greater lean mass provides better balance, but also because greater muscle-tendon traction on the bone promotes an osteogenic stimulus. These results suggest that an impact training (Nikander et al., 2012) seems to have a higher impact on bone structural strength and distribution that aerobic training (Irwin et al., 2009).

Considering biomarkers of bone metabolism exercise can modulate those intervening on osteogenesis (Knobf et al., 2016; Kim et al., 2016; De Paulo et al., 2018). Investigating the physical activity relation with bone turnover biomarkers, it has been showed that the women who perform regular light exercise have high levels of Serum collagen type I amino-terminal propeptide, that is a bone formation marker, and low levels of Tartrate-Resistant Acid Phosphatase 5b, a bone absorption marker (Saito et al., 2020). Moreover, both bone resorption biomarkers (amino-terminal N-telopeptide of type I collagen and Osteocalcin) have shown an increase in those women undergoing hormone therapies performing physical activity in comparison to untrained control groups (Knobf, M.T., 2008; Winters-Stone et al., 2011; Knobf et al., 2016; De Paulo et al., 2018). The results of the studies reported in Table 2 highlighted the positive effect of exercise on stimulating bone remodeling markers, but it is important to underline that the type and the lasting of training stimulus it is a central point to directly affect BMD in premenopausal and postmenopausal women undergoing treatments. In fact, although a positive impact on bone remodeling biomarkers may be obtained after just 6 months of exercise, if the physical activity protocol lasts <9/12 months no BMD increase seemed to be induced (Knobf et al., 2016; De Paulo et al., 2018). Moreover, according to the studies investigated, walking (Knobf, M.T.,) seems to not have the same positive effect on bone metabolism of an impact or combined training (De Paulo et al., 2018; Winters-Stone et al., 2011).

To date supplementation therapy, vitamin D and calcium, in secondary osteoporosis management is a common preventive approach (Camacho et al., 2020) and it seems to have a positive impact on bone health, despite the studies are still scarce on BC patients. According to the results reported in Table 2, it seems that supplementation of vitamin D and calcium, when combined with exercise, has a greater impact on BMD and biomarkers of bone health than when they are prescribed alone (Waltman et al., 2010; Knobf et al., 2016; Kim et al., 2016; Ashem et al., 2019). Moreover, when osteoporosis' pharmacological therapy of bisphosphonate is prescribed and coupled with well-tailored exercise protocol seems to improve its osteogenic effect more compared to the bisphosphonate alone (Waltman et al., 2010). This result suggests that coupling exercise and pharmacological therapy, as bisphosphonate, or to supplementation such ad vitamin D and calcium might lead to better results in BMD management in BC women. Even when the exercise proposed is an innovative protocol, still under investigation, such as pulsed electromagnetic field stimulation (Yuan et al., 2018; Ashem et al., 2019). It is still not clear if this type of training can have the same effect on bone health than a more ‘conventional’ training, such as impact and resistance protocols. Furthers investigations are needed to deeply understand the precise frequency, intensity, type, time of protocol able to counteract bone loss in ER + BC women.

## 6 Conclusion

This review highlights that combining a well-tailored supervised exercise with supplementation (such as Vitamin D and Calcium) and pharmacological therapy may maintain leads to better results in terms of osteogenesis and bone health, reduction of bone loss, promotion of bone turnover biomarkers (Osteocalcin, 25-Hydroxyvitamin D) and decrease of bone resorption biomarkers (amino-terminal N-telopeptide of type I collagenNTx). To overcome the lack of exercise guidelines about bone health management in ER + BC patients under hormone therapies specifically with AIs, this review analyzed all trials on this topic.

According to the scientific evidence concerning physical activity in bone loss prevention, the recommended exercises must mainly involve those muscles that originate and are inserted in the anatomical sites where osteoporosis most frequently occurs (hip, femoral neck, and lumbar spine) to activate the dynamic response of the bone (Frost, 2003; Maycas et al., 2017) due to the exerted tendons' tension. To promote that, the most effective exercise protocols affecting bone health seem to be resistance training (Waltman et al., 2010; Winters-Stone et al., 2011; Thomas et al., 2017; Yuan et al., 2018). Even Ashem et al.'s exercise protocol, based on pulsed electromagnetic field stimulation, shows great efficacy in bone metabolism, but it is currently too early to draw definitive conclusion. Following the results of the studies examined in Table 3, and in order to improve the existing guideline, we summarized which may be an effective training methodology to suggest as integrative therapy in BC patients undergoing AIs (Table 3). Resistance training should be performed 2/3 times a week lasting 45–60 min at a workload of 60/80 % 1RM for 2/3 sets of 6/8 repetitions, or bodyweight exercises with 10 repetitions for 10 sets.. Moreover, the duration and frequency of the exercise intervention play an important role: 12–24 months of training with a frequency above ≥2 sessions of 1 h per week seems to be the best indication to achieve results on BMD (Table 4).

**Table 4:** Practical Application of Physical Exercise. Abbreviations: Reps, Repetition; RM, Repetition Maximum; BMD, Bone Mineral Density; min, minutes; *sec*, seconds.

Practical application				
Type	Day/week	Duration	Intensity	Recommendations
Resistance training	2/3	45/60 min	2/3 sets of 6/8 reps 60/80 % RM	Focus on lower limbs and core muscles
High Impact Exercise	1	10/20 min	Interval training 30s/1min and 1/2 min rest 5/10 sets	Found the appropriate exercises such as steps, hoops, and jumping jacks.
Exercise intervention should last at least 24 months with a frequency above ≥2 sessions of 1 h per week in order to achieve results on BMD.				

Further studies should be carried out to analyze the effect of the intervention protocols suggested above, in order to establish adapted exercise as a complementary preventive therapy in women ER+ BC undergoing AIs treatment.

## Funding

This research did not receive any specific grant from funding agencies in the public, commercial, or not-for-profit sectors.

## CRediT authorship contribution statement

**Claudia Cerulli:** Writing – original draft, Resources, Conceptualization. **Elisa Moretti:** Writing – original draft, Resources, Conceptualization. **Elisa Grazioli:** Writing – review & editing, Visualization, Supervision. **Gian Pietro Emerenziani:** Writing – review & editing, Visualization. **Arianna Murri:** Visualization, Resources, Methodology. **Eliana Tranchita:** Writing – review & editing. **Carlo Minganti:** Methodology, Data curation. **Alessandra Di Cagno:** Methodology, Investigation. **Attilio Parisi:** Writing – review & editing, Visualization, Supervision.

## Declaration of competing interest

All authors have read and approved the final version of the manuscript and agree with the order of presentation of the authors. All the authors have no conflicts of interest to disclose.

## Data Availability

No data was used for the research described in the article.
